# Being Perceived as a Vital Force or a Burden: The Social Utility-Based Acceptance/Rejection (SUBAR) Model

**DOI:** 10.3389/fsoc.2024.1369092

**Published:** 2024-08-05

**Authors:** Michael Dambrun

**Affiliations:** ^1^Université Clermont Auvergne, Clermont-Ferrand, France; ^2^Department of Psychology, University of Calgary, Calgary, AB, Canada

**Keywords:** burden, social rejection, social utility, stigmatization, SUBAR, vital force

## Abstract

This paper proposes a new theoretical model to explain the acceptance/rejection of *agents* (co-workers) and various social groups (people with mental disorders or disabilities, the elderly, the unemployed/poor, ethnic minorities) in a given social system: the social utility-based acceptance/rejection (SUBAR) Model. Based on a social utility approach, it is proposed that human social cognition evaluates and reacts to agents/groups in a social system on the basis of the perceived strengths and significant contributions they bring to the system (*upward forces*; e.g., skills, resources, willingness) and the perceived weaknesses that may harm the system (*downward forces*; e.g., use of social benefits, dependence). While the perception of upward forces for the system (i.e., *vital forces*) is accompanied by acceptance (positive attitudes and behaviors), the perception of downward forces (i.e., *burdens on the system*) promotes rejection (negative attitudes and behaviors). The combination of the two indicators predicts that low vital forces/high burden targets will be the most rejected and high vital forces/low burden targets will be the most accepted. The high burden/high vital forces and low vital forces/low burden targets should be evaluated at an intermediate level between the other two. This naive calculation of the forces exerted by agents/groups in a social system is moderated by various variables (scarcity of economic resources, values) and responds to a functional attempt to regulate individual and collective interests, themselves dependent on the efficiency of given systems. Finally, the relationship of the SUBAR model to other relevant theories will also be discussed.

## Introduction

There is a rich social psychology literature on the variables and processes that promote *positive* (acceptance) and *negative* (rejection) attitudes and behaviors toward others. In this theoretical paper, we propose that the valence of attitudes and behaviors toward “agents” and various social groups (e.g., the mentally disordered, the disabled, low SES/social class groups, the elderly, ethnic and minority groups) is, at least partly, based on their perceived “social utility.” The proposed model provides a theoretical framework that contributes to explaining acceptance/rejection at the interpersonal level (e.g., ostracism) as well as at the intergroup level (e.g., prejudice, discrimination).

The idea that social utility plays a role in social judgments is not new, particularly with respect to the use of personality traits (e.g., Beauvois and Dubois, 2009). We will propose a model in which the perception of social utility shapes attitudes and behaviors toward agents and/or members of social groups in given social systems. Social utility is understood here as a core multidimensional social perception (i.e., material/economic, community, interpersonal utility) that drives social evaluation. This aspect will be elaborated upon further in the “social utility” section. Our social utility-based acceptance/rejection (SUBAR) model will postulate that human social cognition evaluates and reacts to, at least in part, agents/groups in a social system on the basis of the perceived strengths/contributions (“upward forces”; i.e., skills, resources, willingness)—the “vital forces” dimension—they bring to a system and the perceived weaknesses (“downward forces”)—that may harm a system—the burden dimension. A *system* is here understood as a social system, which is a structured network of relationships between individuals, groups, and institutions. This may concern a small group (a dyad, work team, family) or larger social groups, such as communities, cities, nations, companies.

## A model of acceptance/rejection of individuals and groups based on social utility

### Social utility is underlined by two main dimensions: vital forces and burden

**Figure 1:**
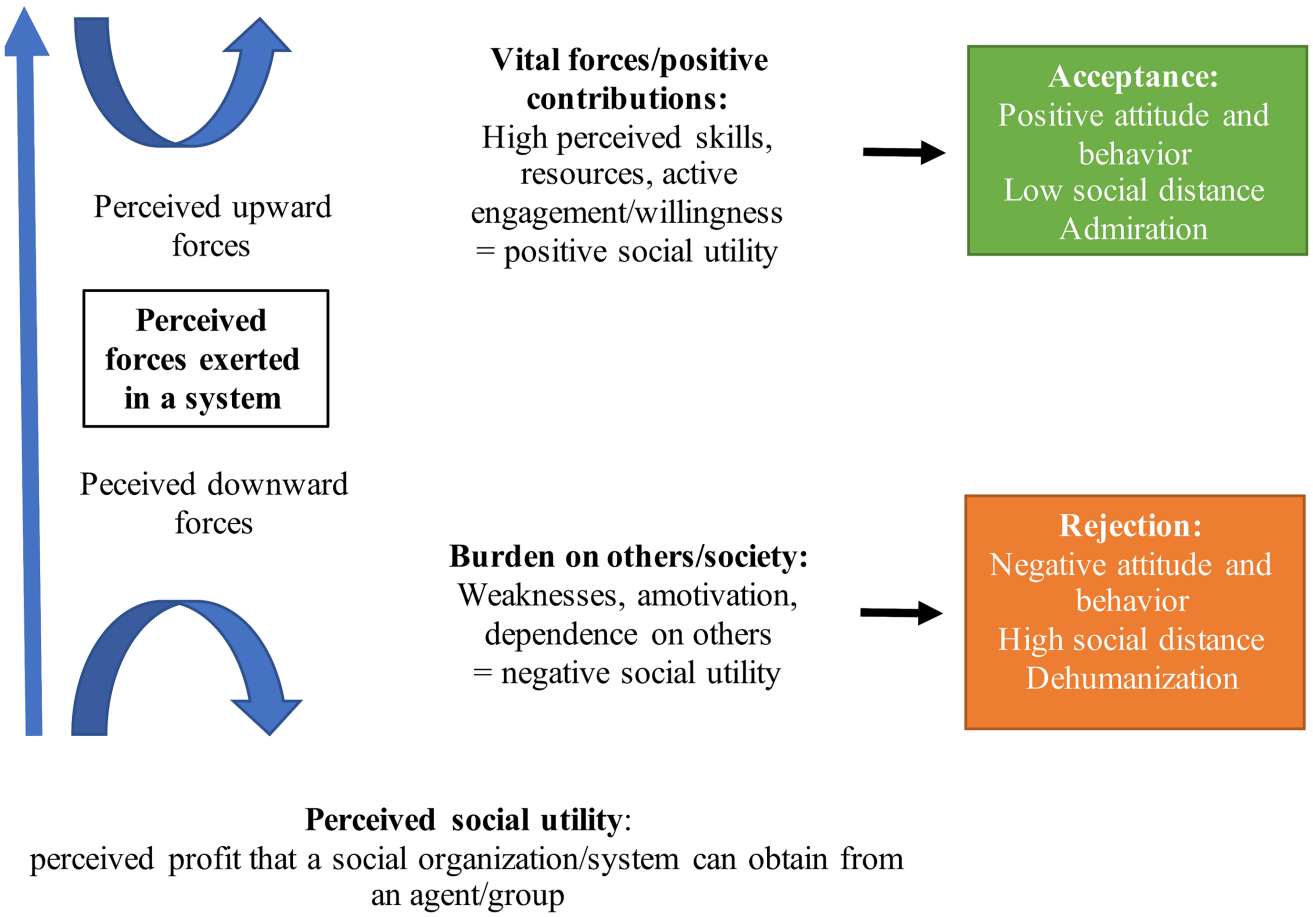
The social utility-based acceptance/rejection (SUBAR) model.

Our SUBAR model begins with the premise that when belonging to a group or organization of any size (e.g., a group of workers, society), individuals basically dichotomize two antagonistic forces (see Figure 1). On the one hand are the *upward forces*, which are made up of all the properties of individuals or groups that add value to a system (i.e., the *vital forces*, the positive contributions), that make a system better or more efficient in the creation of resources with a positive social value. This can be the skills of individuals or groups; their psychological, physical, or material resources that they put to the benefit of the system; and their capacity and active engagement (i.e., their *willingness*) to create wealth. Efficient, dynamic, innovative are characteristic traits of individuals/group members perceived as exerting upward forces.

On the other hand, a system contains *downward forces*, which are made up of everything that constitutes a weakness that can harm the system and weigh it down (i.e., the burdens on a system). Individuals and/or groups who are supported by social benefits and public money may be perceived as both exerting a downward force on the system and burdening society. At another level, those who are overly dependent on others in an interpersonal or family context can be perceived as a burden on the caregiver, for example. Personality traits such as lazy, idle, or vulnerable would belong to this dimension.

Of course, upward and downward forces are *perceptions* that do not necessarily reflect reality. What is valued as resources for an organization may depend, at least in part, on the culture of any given organization. While money or material wealth is undoubtedly a positive value in many organizational cultures, one can imagine that wisdom or ethics may vary significantly. We will not go into detail here about the important variations that can exist at this level. We will limit ourselves to considering that, regardless of organizational culture, variations are always resources that have a potential positive social value (Sidanius and Pratto, 1999), and perceptions of vital force versus burden would be organized around those resources that are perceived as having a positive social value in a given social and cultural system/context.

### Perception of vital force/burden and acceptance/rejection of agents/groups

The core of our SUBAR model is the proposition that the valence of attitudes and behaviors (acceptance/rejection) toward agents/groups in a given social system is directly related to the perception of upward and downward forces possessed by those agents/groups. Hypothetically, while the perception of *significant* (exerting) upward forces would generally be accompanied by acceptance (i.e., positive attitudes and behaviors), the perception of significant downward forces would generate rejection (i.e., negative attitudes and behaviors). More precisely, the combination of the two perceived exerted forces would result in four possibilities (Table 1). The most unfavorable would be the one that combines low vital forces with high burden. This could be the case for agents or groups who are perceived as making no positive contributions to society and who are dependent on social welfare, public finances, or others (e.g., the poor, the disabled, or those who have a mental illness such as addiction or schizophrenia, the elderly, disadvantaged ethnic minorities). The most favorable combination would be one that involves high vital forces and low burden. This refers to agents/groups who are perceived as making a positive contribution to society and who do not exert any constraints that would weaken collective resources (e.g., medical professionals, Nobel prize winners, job-creating employers, firefighters). These “social targets” would elicit admiration. Then there would be two intermediate combinations, including first, weak vital forces and low burden. These would pertain to agents/groups who are perceived as having a relatively neutral contribution, who lend no real vitality to the system, but do not weigh down the collective, either (e.g., most “standard” employees). Then, and finally, there would be the combination of vital forces with high burden. In this case, there would be the perception of an important contribution to the system, but one that would be diminished by an important cost to the collective (e.g., a civil servant, or those with certain mental disorders who cost the State money but with special abilities that provide vitality, as in some forms of autism or entrepreneurs who contribute their know-how but who shy from sharing their material wealth).

**Table 1:** Combinations of vital force and burden perceptions.

	**Perceived upward forces** Vital forces/contribution dimension	
	Low	High
**Perceived downward forces** Burden dimension		
Low	Neutral attitudes/behaviors (most employees)	Strong acceptance/Positive attitudes and behaviors (Agents/groups exceeding standards, high prestige; e.g., medical professionals, Nobel prize winners)
High	Strong rejection/ Negative attitudes and behaviors (Agents/groups perceived as a burden, with a cost; e.g., those who receive social benefits, people with mental illness such as addiction or schizophrenia, the disabled, disadvantaged ethnic minorities, the elderly)	Neutral to middle rejection (Civil servants, certain mental disorders that cost the state money but with special abilities as in some forms of autism, entrepreneurs who contribute their know-how but do not share their wealth)

**Figure 2:**
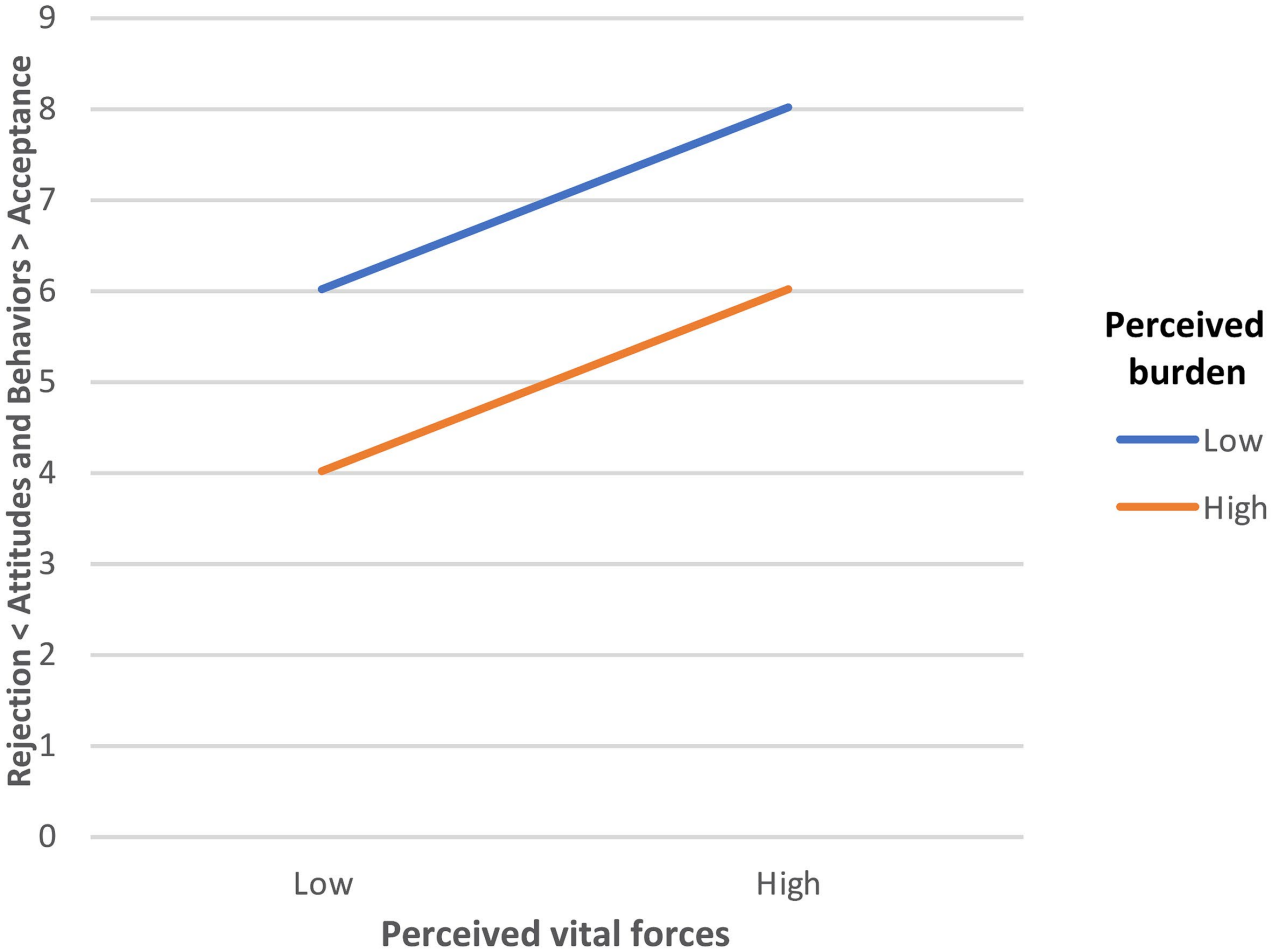
Predicted relationships between perceived vital forces/burden and acceptance/rejection of agents/groups in a social system.

In short, these combinations can be used to predict the level of acceptance or rejection of social targets. This is summarized in Figure 2.

We also propose that some social-psychological variables can enhance the salience of social utility and perceptions of vital forces/burden and hence their use in cognitive and social operations. We focus here mainly on two potentially moderating variables: a system’s economic resources and values.

### Moderators: a system’s economic resources and values

According to the realist theory of group conflict (Sherif et al., 1961; LeVine and Campbell, 1972) and the instrumental model of group conflict (Esses et al., 1998), defensive strategies are more likely to emerge when resources are scarce or the economic context is “gloomy.” In this type of context, there are fewer resources to share, and one of the reasons why individuals become less tolerant (Esses et al., 1998) might be that they perceive certain subordinate groups more as a burden. Thus, we predict that the perception of burden towards certain disadvantaged groups will increase in a context of economic crisis, and this would explain increases in negative attitudes and behaviors usually observed in these contexts.

We also anticipate that perceptions of vital force and burden are linked to social and cultural values. For example, certain personal values in Schwartz’s circumplex model (e.g., Schwartz and Boehnke, 2004) (“universalism” in particular), have been shown to predict prosociality and cooperation in the prisoner’s dilemma paradigm (e.g., Lönnqvist et al., 2013). At the cultural level, while prosocial values are positively related to *horizontal collectivism* (a cultural value emphasizing interdependence), “proself motives” (e.g., competition) are positively related to *vertical individualism*: a cultural value emphasizing the pursuit of distinction and the desire for special status (Moon et al., 2018). In a related field, Goudarzi et al. (2022) highlighted that the neoliberal economic structure shapes a “distributive belief” based on fairness at the individual level. Their findings indicate that neoliberalism has played a central role in shifting distributive justice beliefs from a preference for equality to a preference for merit. Among other psychological effects, neoliberalism promotes an “individualistic-entrepreneurial” self, where the self is seen as autonomous from the social and material environments (Adams et al., 2019), which favors normative meritocracy. Goudarzi et al. (2022) wrote that “neoliberalism encourages the belief that societal benefits and burdens ought to be allocated in proportion to individuals’ contributions to the collective” (p. 1438). Thus, the “calculation” of the social utility of agents/groups in a social system would be reinforced by neoliberal ideology and policies. Overall, these various works suggest that certain social and cultural values and ideologies will *accentuate* the salience and use of social utility in social judgments (e.g., neoliberalism, proself values), while others will *attenuate* them (e.g., values of universalism and interdependence).

Before presenting support for vital forces/burden in the literature, we present some elements concerning social utility, a core variable in our SUBAR model.

## Social utility

Jara-Ettinger et al. (2016) proposed that human social cognition is organized according to a cognitive framework that allows one to infer, for example, the beliefs, desires, character, or competence of others. They call this the *naive utility calculus*, “that is people assume that others choose actions to maximize utilities – the rewards they expect to obtain relative to the costs they expect to incur” (p. 591). This mechanism has implications for different areas of psychology, including social evaluation. This implies, for example, that agents who are perceived to perform helping actions without extrinsic reward will be evaluated more positively than agents who premeditate actions that harm others.

This cognitive approach is compatible with the more general approach of utilitarianism. Although there are many variations of *utilitarianism*, it generally consists of considering that the *morally correct action* is the one that produces the most overall good, which means taking into account the good of others as well as one’s own good (Bentham, 1785, 1789; Mill, 1998). While one finds important discussions about what constitutes “the good” (e.g., Moore, 1903; Hume, 1978), utilitarianism leads to the prediction that there is a positive social value associated with acting in the interest of a global good. According to Bentham (1785, 1789), laws and individuals can be considered good or bad according to their utility; *bad* when they tend to lead to unhappiness and misery and *good* when they promote happiness. The contribution of an individual or a group of individuals to a given system would be associated with a degree of social utility. Social utility is not an objective property, but a subjective experience. It is perceived.

The work of Beauvois and Dubois (2009) in social psychology is a reference concerning social utility and its implications regarding others’ perceptions in the paradigm of impression-formation. These authors proposed that personality traits are evaluative and indicate less what individuals *are* than what their social value *is*. These authors claimed that social value is composed of two dimensions: first, *social desirability*, or the extent to which the observed or anticipated behaviors of an individual are perceived as desirable in a given context; second, what Beauvois (1995) called *social utility*, which reflects one’s perception of a person’s chances of success or failure in a system. Here the term “utility” is not to be understood in its functional sense (e.g., in providing a service), but rather from the quasi-economic perspective of a person’s market value. It indicates the profit that a system might obtain from a given person (Cambon, 2006; Dompnier et al., 2007).

We contrasted this with equity theory that offers an explanatory framework for understanding the motivational processes underlying social utility calculations (e.g., Hatfield et al., 2011). This approach is based on several postulates, including that individuals are jointly motivated by both maximizing the profits they can make from others or from a system and the concern that these profits should nevertheless remain relatively fair and equitable (see also Loewenstein et al., 1989). Otherwise, a rather egocentric drive and a need for justice *co-exist*. Society and groups generally reward members who treat others fairly. As a result, individuals are on average more comfortable when they perceive that the benefits they obtain from a relationship correspond to what they deserve. Guilt and shame emerge when the individual perceives that they are receiving more than they deserve, and conversely, anger, sadness, or resentment emerge in the case of perceived injustice (e.g., Tabibnia et al., 2008). Hatfield et al. (1978) proposed that, in the event of inequity, individuals would seek to reduce their distress by various means to, for example, restore fairness. Perceptions of vital force and burden within a system can be conceived as cognitions that are undergirded by the need for fairness (e.g., Folger and Cropanzano, 1998; Tyler and Smith, 1998). If an individual or group is perceived as taking more than they give (i.e., perceived inequity/unfairness), this would encourage perceptions of burden and rejection. Conversely, when an agent or group is perceived as a vital force contributing significantly to the system, this promotes a sense of fairness/equity and acceptance. Thus, perception of equity/fairness would be intimately related to perceptions of vital force versus burdens.

The concept of utility underlying the SUBAR model aligns with social exchange theory (Blau, 2017; Marinho, 2024). According to this theory, judgments about others are made based on a cost–benefit balance. Potential costs are linked to perceived losses in productivity or system disruption (e.g., using social and/or financial resources without reciprocation, deterioration of interpersonal or community dynamics; Levine and Moreland, 1990). Conversely, benefits are elements that contribute to the growth and/or maintenance of the system (e.g., provision of material or symbolic resources, pro-sociality; Levine and Moreland, 1990). These theoretical elements invite us to extend Beauvois’s (1995) approach and to question the dimensionality of social utility. The dimension, adopted by this author, can be described as material/economic in the sense that the target of the evaluation is judged based on their direct or indirect contribution, constituting a reference related to market value and/or a positive social value, whether symbolic or material (Sidanius and Pratto, 1999). To this dimension, it is proposed to add at least two other aspects. The first is at an interpersonal level. This involves utility in direct or indirect relationships with others, as individuals. For example, the ability to resonate with others in their particularities (empathy, compassion), and to provide help (pro-sociality) are some of the markers of this dimension (related to warmth) on which individuals can base their perception of utility (e.g., Smillie et al., 2019). The second relates more to community aspects. For instance, the ability to contribute positively to relationships between individuals, maintain harmony within a system, enhance the well-being of society, or contribute to community cohesion seem to potentially constitute the perception of social utility at the community level (e.g., Price, 2003).

## Vital forces and burden: support in the literature

Here we review works that have addressed vital forces and burden dimensions in one way or another. We begin with literature that has focused on the characteristics of people identified as vital forces for a society, such as entrepreneurs, leaders, and the wealthy, and how they are perceived by others and how they can elicit admiration. We continue with the concept of burden, which seems to be associated with certain social categories. We also consider both studies on the ostracism of people perceived as deviant and burdensome as well as the psychological effects of feeling a burden to others.

### Vital forces

In the paper *Development and socialization within an evolutionary context: Growing up to become “A good and useful human being*,*”* Narvaez (2013) suggested that *desirability* (being good, appreciated) and utility are two central dimensions for human beings (see also Beauvois and Dubois, 2009). According to sociobiology and evolutionary psychology, a selection process produces and improves characteristics that are useful for survival. Cultural psychology has revealed that *what* is useful in terms of behavior is culturally dependent. A classic study of this phenomenon was provided in 1967 by Berry, where Temne from Sierra Leone and Eskimo from Baffin Island were asked to complete a task assessing their degree of conformity. Berry concluded that subsistence societies tend to produce a degree of conformity in individuals *required* by their economies. There are probably a variety of useful behaviors, and this usefulness probably depends on cultural context, which is beyond the scope of this paper. However, we can examine in the Western context which characteristics of individuals and groups are associated with a positive social value, such as wealth or status.

Some psychologists have tried to identify the characteristics of successful leaders, or the factors that lead to wealth in societies. In their review, Frese and Gielnik (2014) presented meta-analytic findings revealing that personality dimensions such as self-efficacy and need for achievement are highly associated with entrepreneurship. They ultimately proposed a model that included various characteristics such as cognitive abilities (e.g., expertise, practical intelligence), motivational factors (e.g., passion), or action characteristics (e.g., personal initiative) that promote successful entrepreneurship. Some researchers have attempted to identify the personality type of wealthy people, and some traits have been shown to be significantly related to wealth, including conscientiousness, openness to experience, and emotional stability (Mueller and Plug, 2006; Klontz et al., 2014).

More relevant for our paper are several studies that examined the stereotypes associated with these categories. A germane question was: What do people think are the strengths and qualities of wealthy people and leaders? Examining lay explanations of wealth, Forgas et al. (1982) found that participants used four broad categories of attribution to explain wealth: external-social, internal-individual, family background, and luck-risk factors. *Internal-individual factors* were composed of careful money management, good business sense, hard work and effort, intelligence, and risk-taking ability (*cf.* Arnulf et al., 2022). Stoltz (1959) assessed subordinates’ perceptions of “the productive engineer” and found that they were perceived, among other things, as an intelligent person with good analytical skills. Interestingly, Bruce (1994) described a survey indicating that ethical behaviors among productive people is a desirable and important dimension. This is consistent with the idea that strengths alone are likely *not* enough to lead to a positive attitude. Virtuous behavior and respect for the collective interest are likely to be required. Lack of ethics or corruption might be associated with a perception of burden that would attenuate the benefits associated with strengths, making attitudes and behaviors less positive and even very negative in some cases [e.g., corrupt politicians wielding power (Anderson and Tverdova, 2003)].

Other studies have shown that the perception of strength is generally linked to admiration, a positive emotion that has numerous positive consequences for the dynamics of interpersonal and intergroup relations. Onu et al. (2016a) proposed to distinguish admiration from other positive emotions such as elevation, gratitude, awe, envy, and adoration. They defined *admiration* as a human other-focused emotion, elicited by skills exceeding standards that can manifest at various social levels (individual, dyad, group). Admiration elicitors mainly demonstrate above-standard competences (Fiske et al., 2002; Algoe and Haidt, 2009), prestige hierarchies (Henrich and Gil-White, 2001; Fessler and Haley, 2003), and legitimate high status (Onu et al., 2016b). Several works concur in suggesting that admiration is more likely to occur when the target’s—the model’s—position is attainable (Smith, 2000; Schindler et al., 2013). The consequences of admiration are particularly interesting for us. First, at the intraindividual level, admiration elicits energizing sensations and motivations favoring self-improvement and learning (Algoe and Haidt, 2009; Schindler et al., 2015; Onu et al., 2016b). It also improves interpersonal and intergroup relationships as it elicits praise for the admired person/group, willingness to receive learning-related help, and cooperation/contact (Cuddy et al., 2007; Algoe and Haidt, 2009). Finally, at the group/cultural level, admiration facilitates cultural transmission (Henrich and Gil-White, 2001) by inspiring a diffusion of skills. It also promotes group prestige and hierarchy maintenance (Sweetman et al., 2013). In sum, consistent with our SUBAR model, attributing vital forces to individuals or groups should encourage the positive emotion of admiration and social acceptance through positively valenced attitudes and behaviors.

### Burden

It seems that considering certain mental illnesses or disorders as “burdens,” especially economic burdens, for society is widespread, including in the scientific literature. To name a few, some studies have evaluated the burden of dementia (Wolters and Ikram, 2018), Parkinson’s disease (Whetten-Goldstein et al., 1997), migraines (Ferrari, 1998), smoking (Raynauld and Vidal, 1992), post-traumatic stress disorder (Kessler, 2000), personality disorders (Soeteman et al., 2008), generalized anxiety (Wittchen, 2002), or mental disorders in general on society (Ustün, 1999; Trautmann et al., 2016). Soeteman et al. (2008) reported “Some evidence suggests that personality disorders are associated with a high economic burden due to, for example, a high demand on psychiatric, health, and social care services” (p. 259) and “Treatment-seeking patients with personality disorders pose a high economic burden on society, a burden substantially higher than that found in, for instance, depression or generalized anxiety disorder” (p. 259). Another example was provided by Trautmann et al. (2016):

In 2010, mental and substance use disorders constituted 10.4% of the global burden of disease and were the leading cause of years lived with disability among all disease groups. Moreover, owing to demographic changes and longer life expectancy, the long-term burden of mental disorders is even expected to increase. (p. 1245).

These examples illustrate the so-called “economic burden” on society. But another form of burden has been proposed for mental disorders: the burden on families and caregivers (i.e., interpersonal and in group level burdens). For example, Corrigan and Nieweglowski (2019) proposed a U-shaped relationship between familiarity and the stigmatization of mental disorders. The association between high familiarity and stigma would be explained, at least partly, by perceived burden (e.g., burden on a family).

Mental disorders do not seem to be the only social categories to be perceived, or to perceive themselves, as burdens on society. For examples, some studies indicate that poor or low SES individuals (Mishra and Singhania, 2014) or the elderly (Kruse and Schmitt, 2006; Cahill et al., 2009) are vulnerable to similar perceptions.

Social psychology has also examined both the ostracization of people perceived as deviant and burdensome (e.g., Wesselmann et al., 2013) and the psychological effects of “feeling like a burden” on others (e.g., Gorvin and Brown, 2012). To our knowledge, the perception of burden in the eye of the beholder has been studied primarily at the interpersonal level, but much less at the intergroup level. For instance, Wesselmann et al. (2015) manipulated “burdensome deviation” by programming a group member to perform slower than others in a virtual game. Participants perceived the slowest players to be burdensome and ostracized them, but they did not ostracize Goth players who were perceived as deviants but not burdensome (*cf.* Wesselmann et al., 2013). Wirth et al. (2020) examined social costs that result from a poor performance in a group. As anticipated, harmful poor-performing participants felt more like a burden, felt ostracized/rejected, and experienced negative affect (*cf.* Wirth et al., 2015).

To examine the relevance of burden at the intergroup level, we reviewed some of the prejudice scales used in social psychology to assess attitudes toward various social groups. Some scales not only measure the negative/positive valence of attitudes toward a given group but also include certain perceptions. Without being exhaustive, Table 2 presents a list of items related to the concept of burden from different scales. The notion of “burden” was present in several scales assessing racism against various ethnic minorities (e.g., African Americans in the United States or Arabs in France). Most of these items are related to economic burden; the perception, for example, that the target benefits from public money without real compensation (e.g., “X benefits unfairly from other people’s money”). Some items in Pettigrew and Meertens' (1995) scales also refer to a lack of vital strength (e.g., “X comes from the less-able races, and this explains why they are not as well off as most British people”). We also found support for the notion of burden in scales assessing prejudice towards the elderly, the unemployed, and the disabled. Here again, the issue of economic burden comes to the fore for the elderly (e.g., “Older people are too expensive for public budgets”), and also for the unemployed (e.g., “I am shocked if the long-time unemployed have an easy-going life at the expense of society”). Finally, regarding disabled people, both the economic aspect of care (e.g., “Persons with disability usually ask for special treatment for their disability”) and the social aspect of the burden, such as the burden on careers (e.g., “Persons with disability tend to leave difficult tasks for people without disability”), come to the fore. With our SUBAR model in this article, however, we propose to differentiate the perception of burden from prejudice (i.e., negative attitudes) by suggesting that the former is a *cause* of the latter. Consequently, the more a target would be perceived as an economic and social burden, the more they would be rejected at both attitudinal (i.e., hostility, negative attitudes) and behavioral levels (e.g., avoidance, social distance).

**Table 2:** The notion of burden in existing prejudice scales toward various social groups.

Type	Name of the scale	Author(s)	Example of item related to “burden”	Component
Racism	Blatant prejudice	Pettigrew and Meertens (1995)	“Most X living here who receive support from welfare could get along without it if they tried”	Burden
	Blatant prejudice	Pettigrew and Meertens (1995)	“X come from less-able races and this explains why they are not as well off as most British people”	Lack of vital force
	Subtle prejudice	Pettigrew and Meertens (1995)	“It is just a matter of some people not trying hard enough. If X would only try harder, they could be as well off as British people”	Burden
	Subtle prejudice	Pettigrew and Meertens (1995)	“X living here teach their children values and skills different from those required to be successful in Britain”	Lack of vital force
	Symbolic racism	Kinder and Sears (1981)	“Do you think that most X who receive money from welfare programs could get along without it if they tried, or do they really need the help?”	Burden
	Prejudice toward Arabs	Dambrun (2007)	“X living in France do not need to work; the social benefits they receive are more than enough to live on”	Burden
	Prejudice toward Arabs	Dambrun (2007)	“X benefit unfairly from other people’s money”	Burden
Ageism	Measurement of agreement with age stereotypes and the salience of age in social interaction	Kruse and Schmitt (2006)	“Older people are too expensive for public budgets” “The growing proportion of older people undermines our economic competitiveness”	Burden
Prejudice toward the unemployed	Devaluation of unemployed	Hövermann et al. (2015)	“I am shocked if long-time unemployed have an easy-going life at the expense of the society”	Burden
Prejudice toward the disabled	Attitudes and perspectives toward persons with disabilities	Myong et al. (2021)	Fourth factor: “Sense of burdening”: “Persons with disability usually ask special treatment for their disability”; “Persons with disability tend to leave difficult tasks for people without disability”	Burden

Finally, there is consequent literature on the effects of *feeling like a burden* and “self-stigma.” According to Gorvin and Brown (2012), experiencing a sense of being a burden on others has been documented for several groups such as the elderly, people with disabilities, and those with a chronic/terminal illness. McPherson et al. (2007) defined self-perceived burden as an “empathic concern engendered from the impact on others of one’s illness and care needs, resulting in guilt, distress, feelings of responsibility and diminished sense of self” (p. 425). Perceived dependence on others is another important factor to account for (Cousineau et al., 2003). Feeling like a burden can have serious consequences such as poor mental health [e.g., depression, hopelessness, reduced quality of life (Joiner, 2005)], suicidal behavior (Joiner and Silva, 2012), or altered social interactions (Charmaz, 1983, 1994; Galvin, 2005).

Interpersonal acceptance-rejection theory (Rohner, 1986) also allows us to consider the potentially deleterious effect of rejection based on the perception of burden from a developmental perspective. This theory suggests that the perception of being accepted or rejected by one’s parents has direct consequences for psychological adjustment, both during childhood and adulthood (Khaleque and Ali, 2017). For example, the SUBAR model proposes that the perception of a child as a burden (e.g., a difficult child, a child with a disorder or a disability) promotes rejection and, consequently, indirectly leads to deficient psychological adjustments. Perception could be one of the causal factors, similar to social support, which has been identified as a variable favoring the acceptance of children with disabilities by their parents (Gusrianti et al., 2018).

In sum, the concept of burden seems relevant at different levels of analysis, first, at the level of the self, with its consequences in terms of self-stigmatization, then at the interpersonal level, with all that concerns the burden on others (i.e., vis-á-vis a career) and the in group (e.g., one’s nuclear family). The perception of burden also seems to have a stake in the intergroup level, in the perceptions of outgroups, for instance. Finally, the economic level also seems important to consider, which may well cover different levels, such as the financial burden on a family but also broader levels such as the cost to an organization, a society, or a country. However, while the concepts of vital forces and burden in relation to social utility have not been formalized theoretically in the existing literature, several works provide support for this conceptualization.

## Vital forces/burden and the fundamental dimensions of content in social cognition and emotion

### Agentic/communal contents and vital force/burden

A large body of research shows that two main dimensions are involved in the perception of other persons and social groups: the *agentic content* that refers to goal-achievement and task functioning (competence, assertiveness, decisiveness) and *communal content*, which has a social function of maintaining relationships and facilitating positive interactions (e.g., helpfulness, benevolence, trustworthiness). These two dimensions have been called “fundamental” (Fiske et al., 2007; Peeters, 2008; Abele and Wojciszke, 2014), or the Big Two (Paulhus and Trapnell, 2008). Although there are links, our SUBAR model differs somewhat and proposes that what determines whether a target is accepted or rejected in a given social system is not so much their skills (the agentic dimension) or kindness (the communal dimension), for examples, but their perceived “contribution” to the system, which is primarily linked to perceived social utility. In addition, we propose that traits associated with social utility cannot be reduced to the agentic dimension. We suggest that social utility encompasses broader characteristics such as certain dimensions of communality like trust or altruism and sharing. Behaving ethically and in the interests of the greatest number must also be an important aspect (Bentham, 1785, 1789; Mill, 1998). Thus, further refinement is undoubtedly required to properly operationalize this notion.

### Social utility traits and vital force/burden

Some studies have tried to determine which traits best reflect the social utility dimension. Dompnier et al. (2007) asked teachers to evaluate their pupils on 24 personality traits pre-selected from a larger pool of 150 on a scale ranging from 0 (“Does not describe the pupil very well”) to 10 (“Describes the pupil very well”). A correspondence analysis revealed which traits had the highest loadings on the social utility dimension and on a second dimension they called—in reference to the framework developed by Beauvois (1995)—social desirability. Table 3 presents, from the highest to the lowest, the traits that have loaded positively and negatively on the social utility dimension. When we examined the meaning of the traits, four aspects emerged, including *competence* (efficient, intelligent, thoughtful, cultivated), *will/motivation* (determined, voluntary, studious), *amotivation* (passive, lazy), and *weaknesses* (sluggish, weak, inattentive). This seems compatible with the competence component of agentivity. Social utility also seems to be associated with both a strong motivational component and an absence of weakness, the latter of which might overlap with the notion of burden.

**Table 3:** Perceived social utility associated with various personality traits and occupations.

Dompnier et al. (2007)		Le Barbenchon et al. (2005)		
			Useful	Not useful
Traits loading positively on social utility	Efficient (0.84) Determined (0.78) Voluntary (0.77) Intelligent (0.75) Studious (0.74) Thoughful (0.72) Cultivated (0.69)	Desirable	**Traits** Resourceful Reliable Willing Dynamic Energetic **Occupations** Neurologist Surgeon Veterinarian Pediatrician Physician	**Traits** Dizzy Impressive Soft Fragile Emotional **Occupations** Ice cream maker Nurse Nanny Barman Baker
Traits loading negatively on social utility	Sluggish (−0.69) Passive (−0.69) Weak (−0.67) Lazy (−0.62) Inattentive (−0.60)	Neutral in terms of desirability	**Traits** Imperturbable Meticulous Obstinate Tenacious Perfectionist **Occupations** Biochemist Consul Chemist Dentist Lawyer	**Traits** Vulnerable Clumsy Imprudent Naïve Blundering **Occupations** Deliveryman Laborer Grape picker Bodybuilder Sailor

In a similar vein, Le Barbenchon et al. (2005) asked 139 psychology students to evaluate the social desirability (e.g., level of agreement with the item “To have everything to be loved”) and social utility (e.g., level of agreement with the item “To have everything to succeed in your professional life”) of 308 personality traits and 297 occupations. Table 3 presents the traits and occupations as a function of their perceived utility and desirability. Concerning the traits perceived as “socially useful,” once again, a strong willpower and motivation/determination component emerged (willing, dynamic, energic, imperturbable, obstinate, tenacious, perfectionist). Competence did not appear to be an important dimension in this study. The only trait potentially related to this component was “resourcefulness.” Interestingly, “trustworthiness” (reliable), which is more related to the communal component, was present. Concerning the traits that were perceived as the least useful, we found mainly traits related to weaknesses (e.g., dizzy, soft, fragile, vulnerable, clumsy). Concerning the occupations, the most useful-desirable were all medical professions that require high skills but also involve caring for others. In the “useful but with neutral desirability” category, we found certain scientific, law, and medical professions.

**Figure 3:**
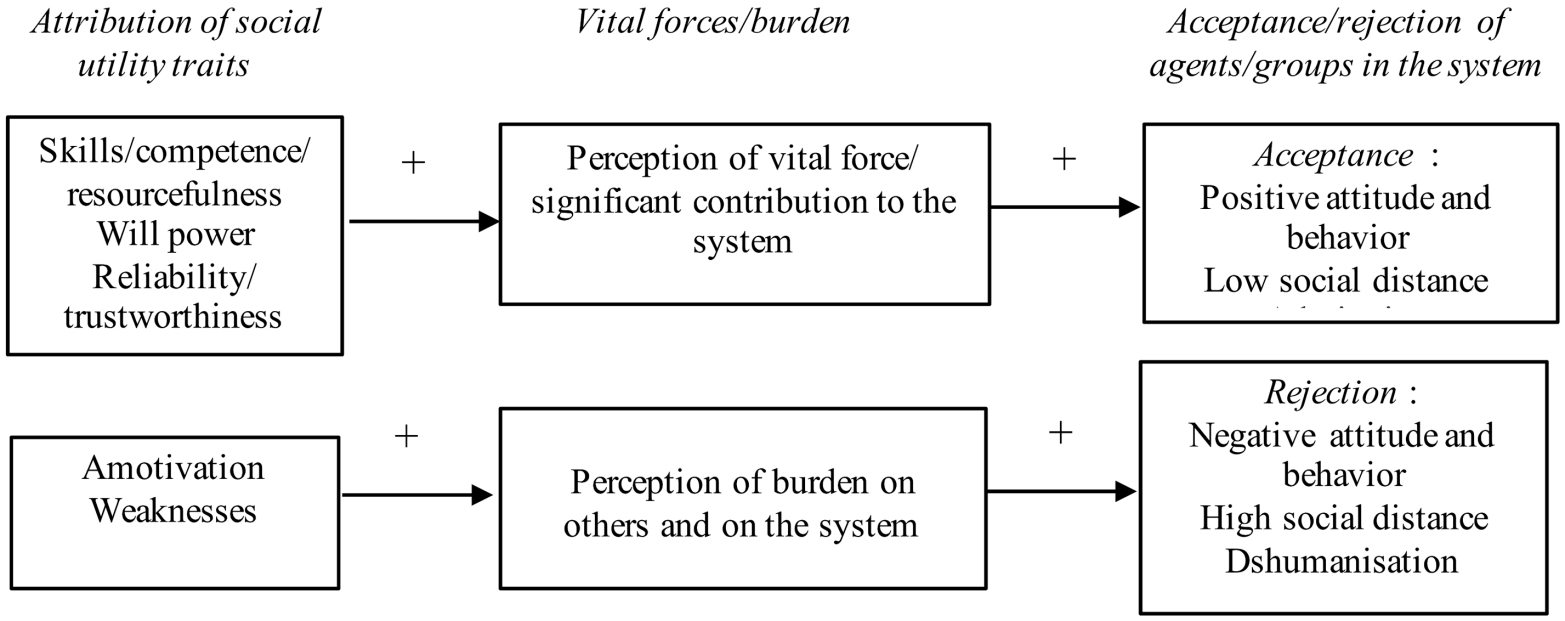
Predictive model of the relationships between traits attribution linked to social utility, perception of vital forces/burden, and acceptance/rejection of agents/groups.

In sum, social utility seems to be associated with a multidimensional content. On the one hand, there are certain traits related to skills, competences, and resourcefulness. Willpower, motivation, effort, and determination to achieve one’s goals also appear as an important dimension. Reliability and trustworthiness also stand out. These traits are quite similar to those we found in the studies presented above on the lay perception of wealthy people and leaders. These traits are probably involved in shaping the perceived vital force/contribution of an agent or group within a given system. However, social uselessness also seems to be an important component to consider. Amotivation and weaknesses (e.g., vulnerability, fragility) could be constitutive of the perception of burden. These types of traits are often associated, for example, with the elderly, people with disabilities, or poor people (e.g., Nario-Redmond, 2010; Fineman, 2012; Lindqvist et al., 2017). This leads to a predictive model in which the attribution of social utility traits at the interpersonal level or the intergroup level (stereotype) would predict the perception of vital force/burden, which itself would help determine the acceptance/rejection of agents and/or groups in given systems (Figure 3). In addition, while the perception of vital force should mediate the relationship between traits associated with social utility and acceptance, the perception of burden should mediate the relationship between traits associated with social uselessness and rejection.

### Vital force/burden and emotions

It is likely that perceptions of vital force and burden are related to certain emotions. As we saw earlier, attributing vital forces to individuals or groups should be related to positive emotion of admiration. But other emotions are probably involved (e.g., Fiske et al., 2002). For example, it has been shown that when an upward social comparison (i.e., comparison with someone “better” than oneself) is experienced as threatening to self-esteem, people feel not admiration, but jealousy (e.g., Mikulincer et al., 1989). Fiske *et al.*’s (2002) model predicts envy due to attributions that groups do not deserve their advantaged position. Thus, the perception of vital force should only arouse admiration when individuals/groups do not pose a direct threat to self-esteem and are perceived as deserving of their advantageous vital forces. If this were not the case, it would rather arouse jealousy and even disdain (Corrigan et al., 2021).

The perception of burden should also be linked to certain emotions such as anger, shame, fear, contempt, and sometimes more positive emotions such as empathy and compassion. The psychology of deviance has shown that individuals can feel different emotions when faced with a person or group perceived as deviant and, sometimes, burdensome: anger (Schachter, 1951; Heerdink et al., 2013), shame and embarrassment (Chekroun and Nugier, 2011), or fear, the latter being particularly present in high authoritarian people (Butler, 2009). Contempt is more likely to occur when disadvantaged individuals/groups are assumed to deserve their unfavorable position (Sadler et al., 2015). However, when they are seen as undeserving of their unfortunate/unjust position, emotions such as pity (Lantos et al., 2020), empathy (Shen et al., 2013) and sometimes compassion emerge (McCall et al., 2014). This leads to the hypothesis that perceived burden would be associated with contrasting emotions depending on perceived fairness. In cases where the burden is perceived as unfair, as in cases where the individual/group is perceived as being the victim of factors that are imposed on them, for example (e.g., PTSD, abusive licensing, harassment leading to burnout), we might expect positive emotions such as empathy or compassion. On the other hand, when the burden is perceived as unfair to others and/or to the group/organization, the result would be anger to signal to the individual or group that they need to change their behavior (Fridlund, 1994; Van Kleef, 2009), in some cases, anticipatory fear, particularly when it is perceived that the situation will get worse and be too costly for others and for the social system, and contempt. Thus, depending on various factors, perception of vital force and burden will be related to distinct emotional patterns. These are summarized in Table 4.

**Table 4:** Perceived vital force/burden and emotions.

	Vital force		Burden	
	Self-esteem threat and/or perceived undeserved vital force	Vital force perceived as fair/deserved	Unfair to the person/group itself	Unfair to others, to the group or organization
Emotions	Jealousy Disdain	Admiration	Pity Empathy Compassion	Anger Anticipatory fear Contempt
Attitudes and behaviors toward the target	Reduced acceptance	Acceptance	Acceptance	Rejection

In addition, the theory of intergroup emotions (Mackie et al., 2008) proposes that emotional reactions to outgroup members condition approach or avoidance behaviors (Frijda et al., 1989). Anger towards an outgroup increases the desire for confrontation or aggression, fear and contempt make avoidance and social distance more likely, while admiration increases approach and imitation tendencies. As a result, emotions may favor acceptance or rejection, and would mediate the relationships between perceptions of vital force/burden and acceptance/rejection.

## The function of vital forces/burden and its relationship to other theories in social psychology

With SUBAR we propose that perceptions of life force and burden have, at least in part, a functional and adaptative origin. It has been proposed, for example, that the agentic and communal dimensions that organize human cognition reflect evolutionary pressures. For instance, Fiske et al. (2007) proposed that competence and warmth reflect “presumed intentions” (hostile or benevolent in the case of warmth) and the ability of others or different groups to realize them (i.e., competences). Peeters (2001, 2008) proposed that the evaluative meaning of traits reflects their adaptive potential to humans. They distinguished between “self-profitable” versus “other-profitable” traits. The former are traits directly beneficial to the person themselves, such as being competent, as this enables the person to succeed in life. The second are traits that benefit others, such as warmth. Starting from the premise that members of a given social system (e.g., family or work team, groups in society) are *not* independent of each other, we suggest that *what* is perceived as being useful to others will depend on both context and perceived interdependence. While the communal dimension undeniably represents a benefit to others (Abele and Wojciszke, 2014), other dimensions linked to social utility would also be relevant.

Collective performance and intelligence, as examples, have been found higher when members have higher average individual intelligence, individual skills that increase the likelihood of collaborating effectively, and skills that facilitate member trust and motivation (e.g., Dirks, 1999; Woolley et al., 2015). Thus, such vital forces should be valued and perceived as other-profitable in a co-worker or group working context, for instance. Also, informal and family caregiver burden is known to impact carers’ mental health, quality of life, and family balance (e.g., Alves et al., 2019; Gérain and Zech, 2021). So, the perception of weakness and apathy can have direct anticipatory negative consequences for others. Individuals should therefore devalue them and perceive them as “other non-profitable” (Corrigan and Nieweglowski, 2019). Moreover, the balance of a country’s spending and wealth has a direct impact on the “common goods.” Individuals in a society therefore should have an interest in associating with and accepting vital forces (i.e., they are other-profitable) and rejecting those who are perceived as making no contribution or taking advantage of the collective wealth without compensation (i.e., they are non-profitable). Thus, the perception of social utility seems to help people manage and balance their personal and collective interests within a given social system.

Finally, social dominance theory can help readers understand the broader place and function of life-force/burden perceptions within the functioning of social systems; this theory, developed by Sidanius and Pratto (1999), postulates that humans have a natural tendency to form hierarchical organizations, and that they develop ideologies that help justify them while enabling the status quo. This theory proposes the existence of two types of legitimizing myths: those that accentuate social hierarchy (racism, sexism, etc.) and those that help to attenuate it (universal human rights, universalism, etc.). Ideologies thus serve to maintain the social system. The main empirical contribution of this theory probably lies in the concept of *social dominance orientation* (SDO), which is defined as the degree to which individuals desire and support social hierarchy, the dominance of subordinate groups by dominant groups, and social inequalities. Consistently, numerous studies have revealed that SDO correlates positively with a variety of “accentuating myths” [e.g., anti-black racism, sexist attitudes, political conservatism, nationalism (Pratto et al., 1994)] and negatively with so-called “attenuating myths” [e.g., gay rights, women’s rights, pro-environmental policies (Pratto et al., 1994)]. What place might the social utility of individuals and groups occupy within this theoretical approach? First, it is likely that SDO is positively related to the perceived vital force of high-status groups and to the perceived burden of subordinated groups. Second, social dominance theory [see also system justification theory (Jost and Banaji, 1994)] proposes that disadvantaged groups participate in their own domination by endorsing ideologies that accentuate hierarchy. From this perspective, it is likely that disadvantaged group members internalize to some extent the perceived burden of low-status groups, particularly when they have a high propensity for social dominance (Jost and Thompson, 2000).

## Existing empirical support for the SUBAR model

A recent preliminary study provides initial support for the SUBAR model (Dambrun et al., 2024). In this study, just over 900 participants completed an online questionnaire assessing various social perceptions, including vital forces and burden, warmth/competence, and dangerousness towards individuals with mental illnesses (e.g., addiction, schizophrenia, depression, anxiety, PTSD, etc.). The questionnaire also included measures of social distance and negative feeling towards the same targets.

This study contributes several findings. First, it provides, for the first time, a map of perceptions of vital forces and burden for the fifteen mental illnesses investigated. Three clusters emerge: illnesses perceived as combining low vital force and high burden (i.e., alcohol addiction and schizophrenia), those perceived with high vital force and low burden (e.g., bulimia, anorexia, ASD, anxiety), and an intermediate group characterized by low vital force and an intermediate level of burden (e.g., burnout, bipolar disorder, depression).

Second, as predicted by the SUBAR model, perceptions of vital force and burden were significantly related, in the expected direction, to measures of social distance and negative feeling for the majority of the mental illnesses investigated. Interestingly, providing incremental validity, these relationships persisted even when measures of danger, warmth, and competence were statistically controlled. The changes in R-squared between the baseline model (i.e., perception of danger, warmth, competence) and the tested model including the perception derived from the SUBAR model (i.e., baseline model + perceptions of vital force and burden) increased significantly in 100% of cases for social distance and 67% of cases for negative feeling.

Of course, these initial results should be interpreted with caution, and further studies are needed. For example, replications involving targets other than mental illnesses, with more heterogeneous and culturally diverse samples, would be welcome. Despite these limitations, these results seem to provide initial incremental validity to this new model.

## Conclusion

With our SUBAR model we propose that calculating the social utility of agents/groups within a social system is central to understanding their acceptance/rejection. Acceptance would be facilitated by the perception of vital forces or significant contributions to the system, while perceptions of burden would favor rejection. It is proposed that these relationships would be moderated by variables such as the scarcity of economic resources and certain values and ideologies (e.g., neoliberalism, prosocial values). The social utility calculus is interpreted as a functional and therefore adaptive attempt to manage individual and collective interest in a social system. The SUBAR model offers a new theoretical framework that can be tested empirically. We hope it will provide a better understanding of the acceptance and rejection of individuals and groups within social systems. It can be applied to a broad range of individuals and social categories and might be useful as a framework for research in areas such as interpersonal and intergroup rejection, and self-stigmatization.

For example, this model could help explain current phenomena such as political extremism. Several authors propose a link between neoliberal ideology and phenomena such as extremism, populism, and right-wing voting (El-Ojeili and Taylor, 2020). We previously discussed the connections between neoliberalism, the prominence of social utility, and its use in social judgments. It is possible that this is involved in certain phenomena, such as right-wing voting, which endorses forms of resource allocation based on merit and the stigmatization of groups perceived as exploiting the system (i.e., as burdens).

Finally, the SUBAR model is likely based on a self-centered functioning guided by the hedonic principle. In this type of functioning, individuals primarily seek to obtain pleasure by accumulating material and/or symbolic stimuli that are pleasant and/or socially valued, while avoiding unpleasant and/or socially devalued ones (Dambrun and Ricard, 2011). Analyzing social relationships in terms of a cost/benefit balance aims to maximize benefits and minimize costs, reflecting a primarily self-interested approach. The SUBAR model is likely to be less relevant when people adopt a more selfless functioning, based, for example, on social harmony.

## Author contributions

MD: Conceptualization, Investigation, Writing – original draft, Writing – review & editing.
